# Subclavicular Dislocation of the Humerus—Open Arthrotomy—Recovery

**Published:** 1891-07

**Authors:** John Parmenter

**Affiliations:** Professor of Anatomy in the Medical Department, University of Buffalo; Surgeon to the Fitch Hospital; Assistant Surgeon to the General Hospital; 372 Franklin Street


Buffalo Medical and Surgical Journal

VOL. XXX. JULY, 1891. NO. 12.

### Original Communications.

### SUBCLAVICULAR DISLOCATION OF THE HUMERUS—OPEN ARTHROTOMY—RECOVERY.[1]

BY JOHN PARMENTER, M. D.,

Professor of Anatomy in the Medical Department, University of Buffalo; Surgeon to the Fitch Hospital; Assistant Surgeon to the General Hospital.

THE rarity of the form of dislocation under consideration, and the unusual method of reduction, are my reasons for intruding upon you so old a subject as dislocation of the shoulder joint.

This joint—a typical ball and socket joint—depends for its integrity, not upon bony conformation, nor upon strong ligaments, but rather upon the muscular attachments connecting the humerus with the trunk. This dependence upon muscles, together with such conditions as a shallow glenoid fossa, a disproportionately large humeral head as compared with the fossa, a weak capsular ligament, and the extensive movements of the arms, combine to make the joint more or less insecure, and more prone to dislocation than any other in the whole body.

The subclavicular variety belongs to the class of forward dislocations, and is one of very great rarity, many of the cases described under this head being really subcoracoid. The head escapes underneath the coracoid process, and can be found under the clavicle and in close relation with its inferior edge. It is quite essential to remember this in making a differential diagnosis between these two forms of dislocation, which in other respects very closely resemble each other. The head and neck lie behind the pectoralis minor, the short head of the biceps, and the coraco-brachialis muscles, or sometimes between these muscles and the serratus magnus. The deltoid, supra- and infra-spinatus, and the subscapularis are put in a condition of more or less extreme ten-

1. Read at the April, 1891, meeting of the Buffalo Medical and Surgical Association.

sion, the tendons of all (the deltoid excepted) being in some cases completely torn across. The damage done to contiguous parts may likewise be great. The axillary nerves may be pushed ahead of the bone, the capsule extensively torn, and the circumflex nerve so injured as to paralyze the deltoid. Such accidents, however, are not common, and in this respect the *forward* are less serious than the downward dislocations. On the other hand, let me add, parenthetically, the forward dislocations, especially the subclavicular, as a class, are distinctly more difficult to reduce than the downward variety. The reasons for this are not always easy to give; but, perhaps, the following explanation may not be far amiss. Soon after the accident, the muscles about the joint have become more or less firmly contracted, thus pulling the head of the humerus closely against the clavicle. When attempts at reduction are made, the great resistance of the muscles and the impinging of the humeral head against the coracoid process combine to prevent the head from entering the glenoid cavity. Add to this the condition of "button-holing," as it is called, where the head has slipped through a small rent in the capsule, and the latter becomes firmly applied to the neck of the humerus, and the difficulties of the case become very much increased. To be sure, this "button-holing" may occur in other shoulder dislocations, but the capsule is considerably more resisting toward the front than downward, and, therefore, is not so liable to be extensively torn as in dislocations into the axilla. Furthermore, the untorn and posterior part of the capsule may add to the resistance in the forward dislocation; so that, when one or more of these conditions exist, we find forward dislocations oftentimes presenting great difficulties in the way of reduction. Do not think, however, that all forward dislocations are so refractory. Many are as easy of reduction as any other form, and this is as true of the subclavicular as of the subcoracoid variety. Bostock reduced a subclavicular luxation of eight years' duration within fifteen minutes, and without an anesthetic.

Let us now suppose that our diagnosis has been made, and that we are employing the usual methods of reducing shoulder dislocations, such as extension in various directions, and some of the different methods by manipulation—when are we to stop? Or, to put it in another way, what are the contra-indications to too prolonged or violent attempts to restore the head of the bone to the glenoid fossa? We know that many and fatal accidents have resulted from injudicious efforts in this direction; such, for instance, as rupture of the axillary artery or vein, or both; injury to the axillary nerves and to the soft parts, causing either immediate irreparable damage or subsequent destructive inflammation; and, finally, fracture of the neck or shaft of the humerus.

Having in mind the accidents that may occur, we understand as a contra-indication any condition which predisposes to or makes liable the occurrence of such accidents. A moment's thought will suggest some of these contra-indications to us. For instance, old age, with its attendant brittleness of bone and degenerative changes in the arteries, may the more easily make the patient undergo a fracture of the humerus, or rupture of the axillary artery. So, also, Bright's disease, or syphilis, may have caused a chronic endar teritis with atheromatous changes in the arterial vessels, and thus predispose to rupture of the axillary. Chronic alcoholism, for the same reason. Again, the time during which the luxation has remained unreduced may contra-indicate too violent or prolonged, or, if sufficient time has gone by, (say six months, on an average, for the shoulder joint,) any attempt at all at reduction. The above-mentioned conditions, then, are among the chief ones which we must have in mind when choosing how we shall reduce a dislocation of the shoulder, or in deciding how long and with how much force we may safely continue our efforts.

Let me now briefly relate the case under consideration, and apply to it such of the principles as have already been considered and are applicable to the case:

B. M., fifty years old; Irish; laborer. Has for many years been addicted to drink. Admitted to the Fitch Hospital, October 28, 1890, at three P. M. The following history was obtained: One week previously, fell and struck upon *shoulder* (existing mental confusion makes this a matter of conjecture). A few hours later he discovered something wrong with his arm, and sought the advice of a physician, who attempted, unsuccessfully, to reduce the luxation. Two or three days then elapsed, when another physician tried reduction, with the same result. On the day of admission he had been seen by two more medical gentlemen, who used their combined efforts for two hours to put the head of the bone in place, without avail. Admitted to the hospital, my house surgeon tried for nearly an hour to reduce it, and, not succeeding, sent for me. Examination showed a tumor, most prominent about the junction of the middle and outer third of the clavicle, and closely applied to its under surface. The other typical signs of dislocation were also present, except the arm was more abducted and the head of the humerus much more fixed than usual. Diagnosis made, the man's general condition was noted, and revealed evidences of exhaustion, such as weak and somewhat irregular pulse — 100 in frequency; his arteries (radials and temporals) markedly atheromatous, arcus senilis very distinct, and the general evidences of a too liberal and prolonged use of stimulants. He was again chloroformed, and during the next hour I tried every known method of bloodless reduction, including a most faithful use of Kocher's method, which I regard as the one most successful in a given number of cases, as compared with other methods. Open arthrotomy was then determined upon, and for the following reasons: First, the man was becoming exhausted, and needed either speedy relief or to be let alone with a useless arm. (The great deviation of the arm from the normal, and its unusual fixity in this abnormal position, made it certain that he could not use the arm in work.) Second, the condition of his arteries. Third, his being older even than his years; and fourth, the great fixity of the head of the humerus.

An incision, between four and five inches long, was made in the substance of the deltoid, toward its anterior border, to better avoid branches of the circumflex nerve. The capsule reached, a further but fruitless effort was made to get the bone in place. The joint was then opened, and the following conditions found present. The neck was tightly grasped by the capsule, the supra- and infra-spinatus and the subscarpulosis muscles very tense; the long biceps tendon lay tensely stretched across the greater tuberosity, it having been thrown out of its groove. It seemed, thus, that the supra- and infra-spinatus and subscapularis muscles held the head of the humerus tightly pulled against both coracoid process and clavicle, while the biceps tendon strapped it down from in front. The hole in the capsule was enlarged, and an attempt at reduction made. Not, however, until the subscapularis and spinati muscles were divided at their insertions was reduction possible, and then only with difficulty. The wound was dressed antiseptically, no drainage being used, except some superficial catgut strands, on account of the edema about the parts, from the manipulation they had undergone. The case progressed favorably, except for the first few days there was some afternoon rise in temperature and restlessness, due to his alcoholic habits. The parts looked red and swollen, but aspiration gave no evidence of pus, and his speedy recovery under mercurial ointment showed conclusively that none existed. At the end of three weeks, passive motion was commenced until he could use his arm freely in all directions, except raising it to a right angle, owing to paralysis of the deltoid. Whether this was due to the original injury or to the operation, I cannot say positively, but it happens so frequently in injuries about the joint, where no cutting has been done, that I feel quite certain that it was due to the original injury. (I have seen paralysis of the deltoid from a blow received in boxing.) He can now, six months after operation, move his arm in all directions, except such movements as the deltoid executes. There is very little grating in the joint, and in all respects it seems quite normal. As for the deltoid paralysis, his age and habits make it exceedingly unlikely that he will ever be better in this respect. There has, however, been comparatively little atrophy of this muscle, and the compensatory action of other muscles enables him to use his arm to good advantage.

And now a few words concerning open arthrotomy as a method of reduction in luxations, irreducible by other means. What have been the results of the employment of this method? What are the dangers? These are important questions, and must be considered in making up our estimate of the advisability of its employment. For the literature upon this subject I am much indebted to an article in Brun's *Beitrage zur klinischen Chirurgie* for 1889, Vol. IV., page 372, by Dr. O. Knapp, of Stuttgart. He considers four methods of operative reduction—viz., sub-cutaneous division of the soft parts about the head, subcutaneous osteotomy of the head, open arthrotomy, and resection of the head.

Only open arthrotomy concerns us here, interesting as it would be to give his views and statistics upon the other methods. He has collected twelve cases in which the luxation had existed from six weeks to two years. Of these twelve cases, two ended fatally (one from delirium tremens and one from suppuration). Of the remaining ten: in one, there was a pseudarthrosis in the surgical neck; in three, necrosis of the head, requiring subsequent extraction; in four, material functional improvement; while the remaining two were too incomplete to base deductions upon. He compares then the statistics of resection with those of open arthrotomy. In twenty cases, four died and six were incomplete. In the remaining ten cases the results were in some equal to, in others better than those in which arthrotomy had been done. From his study of these various methods and their results, he concludes that subcutaneous section of soft parts, or subcutaneous osteotomy, are both unworthy of general acceptance, notwithstanding the fact that they have, at times, yielded good results; and as regards the other method, that it will be advisable to make open arthrotomy only in recent cases—to resect in ancient and neglected cases.

Lister (*Brit. Med. Jour.*, Jan. 4, 1890,) has reported two cases, one of two and the other of seven months' duration. In both the results were excellent, the patients earning their living by hard manual labor.

König says:

Up to now not much has resulted from attempts at bloody reduction, still it is possible that with a better understanding of the conditions which hinder reposition, the results will be better.

In the last (1890) edition of *Hamilton's Fractures and Dislocations*, Stimson has remarked parenthetically that "open arthrotomy has been practised with a gratifying measure of success, and with antiseptic precautions gives promise of being an accepted method." He relates successful cases of Dr. Heron Watson, who operated four weeks after accident, and Dr. Garmany upon a recent case. (Time not given.) To this list can be added the one detailed tonight. Standard surgical works are quite silent on the subject.

The results, then, it seems to me, while no means perfect, are most encouraging, and justify further careful and intelligent use of the method.

What are the dangers?

Suppuration, either from a lack of proper antiseptics or from the presence of chipped-off fragments in the joints. This has happened in the hands of careful and most experienced operators, (Brun's, for example,) and is, in some instances, presumably due not to infection but to necrosis of fragments, which had been separated from their connections during operative manipulations and have escaped notice. Necrosis of the whole head has also occurred. The breaking up of adhesions and putting the head in a new environment has caused this accident in three cases.

Again, there is danger of anchylosis, which may restrict the motions of the extremity much more than did the original condition.

In a case operated upon with strict antisepsis precautions, thus eliminating suppuration by infection from without, these dangers belong almost entirely to the ancient and neglected cases. In a case, recent as my own, for instance, no adhesions worthy of mention can exist, the glenoid fossa is still normal, likewise the head of the humerus, and hence a good result is rather to be expected than otherwise. It is the only one, as far as I can discover, operated upon so early after dislocation, Watson's case of four weeks, standing being the next earliest.

It was but a few days ago that our President kindly asked me to read a paper this evening, and this fact, together with press of other work, has prevented me from making a very thorough examination of the literature bearing upon the subject. A fairly good search through standard works and journals, including the Index

Medicus, gives only the sixteen cases I have mentioned. If to this we add the one considered tonight, we have seventeen in all.

Such a number is far too small to base positive deductions upon, but, it seems to me, that a successful result in a case like my own, in which so many unfavorable conditions existed, is stronger testimony for the safety and efficiency of the method than can ordinarily be claimed from one case. To my mind it is equivalent in this respect to a number of cases in whom youth and health exist, to aid the surgeon in obtaining a good result.

Regarding open arthrotomy the following conclusions seem proper:

1. Carefully and aseptically performed open arthrotomy is safe and ordinarily efficient.

2. It should be employed only in recent cases.

3. When in any case it does not prove efficient, resection can be done through the same incision.

372 Franklin Street.

